# Etiology of Scarlet Fever

**Published:** 1916-03

**Authors:** 


**Etiology of Scarlet Fever.** Felix Schleisser, Jahrbuch fuer Kin derheilkunde, Sept. 1915. 24-hour bouillon cultures of streptococci were blown into the mouth and nose of 27 macacus rhesus monkeys. 12 developed symptoms of scarlatina and were protected against reinfection. The infection was also transferred from monkey to monkey. The filtrate of the culture did not produce infection, so that it is concluded that the streptococcus is the true cause. (Note: It should not be forgotten that the large spirochete of syphilis was not discovered for many years and it is possible that an unfilterable germ may coexist with the streptococci.—Ed.).

